# Simultaneous detection of methylation and genetic variations of BCR-ABL1 gene by nanopore Cas9-targeted sequencing

**DOI:** 10.1016/j.gendis.2023.101190

**Published:** 2023-12-06

**Authors:** Shuilian Xie, Ying Yang, Junjie Zhang, Menglin Zhu, Wang Li, Mengting Li, Yijian Chen, Hailiang Li, Weidan Lun, Weelic Chong, Shaogui Wan

**Affiliations:** a Center for Molecular Pathology, Department of Basic Medicine, Gannan Medical University, Ganzhou, Jiangxi 341000, China; b Department of Hematology, First Affiliated Hospital of Gannan Medical University, Ganzhou, Jiangxi 341000, China; c Department of Laboratory Medicine, First Affiliated Hospital of Gannan Medical University, Ganzhou, Jiangxi 341000, China; d Sidney Kimmel Medical College, Thomas Jefferson University, Philadelphia, PA 19107, USA

The BCR-ABL1 fusion gene is a driver and hallmark of leukemia and a classic structural variant.^1^ Single nucleotide variations (SNVs) occurring in the ABL1 gene kinase domain (KD)^2^ and aberrant DNA methylation modifications, specifically 5-methylcytosine (5mC) of the BCR gene promoter,^3^ have strong clinical implications, such as tyrosine kinase inhibitor resistance, therapeutic responsiveness, and disease progression. Therefore, detecting the presence of KD region mutations and/or promoter 5mC modifications in the BCR-ABL1 fusion gene may help clinicians formulate individualized treatment regimens for patients with leukemia. Currently, the clinical detection of structural variants, SNVs, and 5mC modifications relies on a variety of independent techniques, and comprehensive techniques that can simultaneously detect all three events in one assay are urgently needed. Here, we report the direct detection of all three events using nanopore Cas9-targeted sequencing (nCATS),^4^ which combines Cas9-mediated target enrichment and the advantages of long-read length and direct sequencing of the Oxford Nanopore Technologies platform.

The genomic DNA of K562 cells (which expresses the BCR-ABL1 fusion gene) was used as the experimental sample in this study. We designed four CRISPR-associated RNAs (crRNAs) to enrich the DNA regions of interest (ROIs) (Fig. S1A; Fig. 1A and Table S1). nCATS specifically enriches native DNA within ROIs from pre-dephosphorylated genomic DNA and then ligates sequencing adaptors to the new cuts with a 5′ phosphate group generated by Cas9 and a deoxyribose adenylate added by DNA polymerases to construct a targeted DNA library for nanopore sequencing (Fig. S1B, C). For the analysis of nCATS data, basecalling is performed by Guppy to generate sequencing data (FASTQ files) from electrical signals (FAST5 files); reads are aligned to the human reference genome (hg38) by Minimap2 (Fig. S1D). The downstream variation analysis mainly focuses on identifying the BCR-ABL1 fusion breakpoint, detecting ABL1 KD variants, and assessing the level of BCR promoter 5mC modification (Fig. S1D).

**Figure 1:**
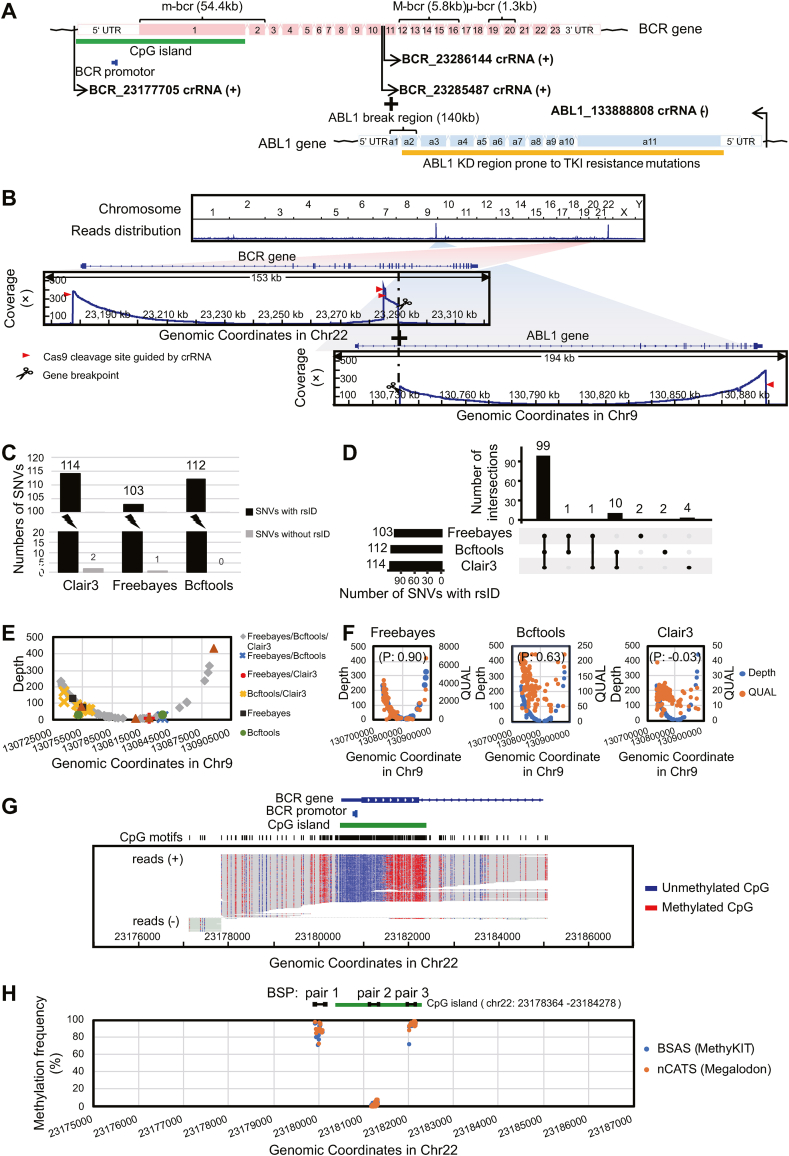
Enrichment design of BCR-ABL1 fusion gene and analysis of nanopore Cas9-targeted sequencing (nCATS) data. **(A)** Structure of BCR-ABL1 fusion gene, and the DNA fragments enriched by the four crRNAs. **(B)** The above image showed the distribution of obtained reads across the human genome and there were distinct peaks of reads found on chromosomes 22 and 9, which, after magnified, were shown in the following coverage plots including the regions of interest enriched by the four crRNAs and breakpoints in the BCR gene and ABL1 gene. **(C)** The bar chart presented all SNVs detected in the ABL1 KD region by Clair3, Freebayes, and Bcftools. **(D)** The UpSet diagram showed the intersection of annotated SNVs with rsID detected by Clair3, Freebayes, and Bcftools. **(E)** The sequencing depth profile represented annotated SNVs with rsID detected individually or jointly by different software. **(F)** Three scatter plots displayed the relationship between the sequencing depth and QUAL value for annotated SNVs with rsID in the ABL1 KD region detected by each of Freebayes, Bcftools, and Clair3. **(G)** Read alignment plots showed methylation patterns around the promoter and CpG island of the BCR gene. **(H)** Methylation calling of the three regions in the BCR gene with high–low–high methylation (BSP pair 1, 2, and 3; approximately 200 bp per region) between nCATS data analyzed by Megalodon and the BSAS data analyzed by MethyKIT. M-bcr, main breakpoint cluster region; m-bcr, minor breakpoint cluster region; μ-bcr, the third breakpoint cluster region; UTR, untranslated region; KD, kinase domain; SNVs, single nucleotide variations; P, Pearson correlation coefficient; BSP, bisulfite sequencing primer; BSAS, bisulfite amplicon sequencing.

To validate the enrichment of ROIs, we analyzed the genome-wide distribution of all quality-controlled reads in our nCATS data. This revealed an apparent peak of reads centered on chromosome 9 (Chr9) containing the ABL1 gene and chromosome 22 (Chr22) containing the BCR gene (Fig. 1B), which were formed by the four crRNA-enriched reads. Through counting these on-target reads, we found that the number of reads covering each of these three ROIs reached approximately 500 (Fig. S2A). In addition, the on-target reads exhibited a typical “NGG” PAM motif end sequence of Cas9 cleavage (Fig. S2B and Table S2). These results indicated a high targeting efficiency of the crRNAs for the ROIs.

When examining the reads enriched by BCR-23285487 crRNA+ and BCR-23286144 crRNA+, we consistently found within each of the reads, a sequence aligned to the 14th intron in BCR main breakpoint cluster region (M-bcr) was immediately followed by the 1st intron of the ABL gene (Fig. 1B). This result indicated that the BCR-ABL1 transcript type and fusion protein in K562 cells were b3a2 and P210^BCR−ABL1^ (Fig. S1A). The breakpoints of BCR and ABL1 were hg38_Chr22:23290555 and hg38_Chr9:130731760, respectively. We further confirmed this BCR-ABL1 fusion gene identified by nCATS using PCR-Sanger sequencing (Fig. S2C).

The identification of SNVs mainly focused on mutations occurring in the ABL1 KD region (exons a2 to a11). We observed that the reads enriched by ABL1_130888808 crRNA− basically yielded complete coverage of the entire ABL1 KD region (Fig. S3). Therefore, we compared and evaluated the SNVs analysis performance of Freebayes, Clair3, and Bcftools in the approximately 157 kb region between the ABL1 gene breakpoint and the ABL1-130888808 crRNA− targeting site. Overall, Freebayes detected significantly fewer SNVs than Clair3 or Bcftools (Fig. 1C and Tables S3A–D). The intersection of annotated SNVs with rsID from these packages showed that Bcftools and Freebayes had an extremely high coincidence, while Clair3 evidently missed about 10 annotated SNVs (Fig. 1D and Table S3D). In addition, because the start site of the on-target reads used to detect the ABL1 KD mutations was fixed and the length was not controllable, whether sequencing depth has an impact on SNV detection needs to be investigated. The results showed that annotated SNVs detected by Bcftools, Freebayes, and Clair3, alone or in combination were randomly distributed and not affected by sequencing depth (Fig. 1E and Table S4A). Furthermore, the relationship between sequencing depth and the QUAL value (an indication of mutation-calling quality) of annotated SNVs detected by each software indicated that only the QUAL value of Freebayes had a very high correlation with sequencing depth (Fig. 1F and Tables S4B–D). Overall, Bcftools had the best performance. Further analysis by Bcftools found that five annotated SNVs occurred in the introns of the ABL1 KD region (Fig. S3), and there were no mutations with known tyrosine kinase inhibitor resistance in the exons in our samples.

The DNA methylation-calling was performed by Megalodon, Python-based software with prominent overall assay performance. We observed that essentially the majority of reads enriched by BCR-23177705 crRNA+ fully covered the CpG island in the BCR gene and the methylation status of C in each CpG motif in this region can be detected (Fig. 1G and Table S5). The methylation status was hypomethylated in the upper half and hypermethylated in the lower half of the CpG island (Fig. 1G). Since the BCR promoter was upstream of the CpGisland, the entire BCR promoter exhibited hypomethylation (Fig. 1G). We verified the 5mC calls with bisulfite-based amplicon sequencing (BSAS) of three representative fragments around the BCR gene promoter and confirmed the high-low-high methylation pattern in these regions (Fig. S4A). The methylation call showed that there was a high consistency with a Pearson correlation coefficient of 0.996 between the 5mC calling results of nCATS based on Megalodon and the sequencing results of BSAS based on MethyKIT software (Fig. 1H; Fig. S4B and Table S6). The above results demonstrated that nCATS was as accurate as the gold standard for detecting BCR promoter methylation, with superior detection range and resolution.

To reduce the complexity of nCATS data analysis, we developed and made freely available an integrative software package “Cas9-nanopore” for the analysis of structural variants, SNVs, and 5mC modifications in nCATS data. The analysis pipeline and the required software in Cas9-nanopore for each variant calling are shown in Figure S5 (see computer availability section for details). Compared with the step-by-step analysis using multiple software packages, the Cas9-nanopore pipeline greatly simplified the process of nCATS data. Furthermore, we successfully validated the utility of the Cas9-nanopore pipeline by analyzing the nCATS data by targeting the human HTT gene, which is a Huntington disease-related gene and is usually used as an internal control in nCATS experiments (Figs. S6A–C and Tables S7A–C).

Overall, by innovating on the nCATS assay, we performed a simultaneous evaluation of the BCR-ABL1 fusion gene, the SNVs of ABL1 KD, and BCR promoter methylation. This comprehensive and efficient detection method is based on long-read sequencing, with many potential clinical applications. Furthermore, this technology is easily adaptable to other genes of interest. In addition, we have made a freely available analysis pipeline for analyzing structural variants, SNVs, and 5mC modifications in nCATS data.

## Author contributions

SW conceived the idea, designed the experiments, revised the manuscript, and supervised the study. SX performed the experiments, analyzed the data, and wrote the manuscript. YY and JZ analyzed the data and revised the manuscript. MZ and ML performed the experiments. WL analyzed the data. YC, HL, and DL prepared the sample and revised the manuscript. WC revised the manuscript. All authors read and approved the final manuscript.

## Conflict of interests

The authors declare no competing interests.

## Funding

This work was supported by the Jiangxi Province's “Double Thousand Plan” Innovation Leading Talent (China) (No. jxsq2019101060), The Youth Jinggang Scholars Program in Jiangxi Province of China, Jiangxi Natural Science Foundation (China) (No. 20232BAB216017), and the National Natural Science Foundation of China (No. 82200653).

## Data availability

The two basecalling FASTQ files of K562 cells' BCR-ABL1 fusion gene and HTT gene are available at The National Genomics Data Center (NGDC), with accession number HRA004394.

## Software availability

The code and usage of the Cas9-nanopore package are publicly available at https://github.com/Center-for-Molecular-Pathology/cas9_nanopore.

## Additional information

Supplementary data (including Methods, Discussion, and Figs. S1–6) and Tables S1–7 of this article are available in separate files.
