# Supplementary figures and images for: Simultaneous detection of methylation and genetic variations of BCR-ABL1 gene by nanopore Cas9-targeted sequencing
Source: Genes Dis. 2023 Dec 6;11(6):101190. doi: 10.1016/j.gendis.2023.101190 (PMC11327521; doi:10.1016/j.gendis.2023.101190)

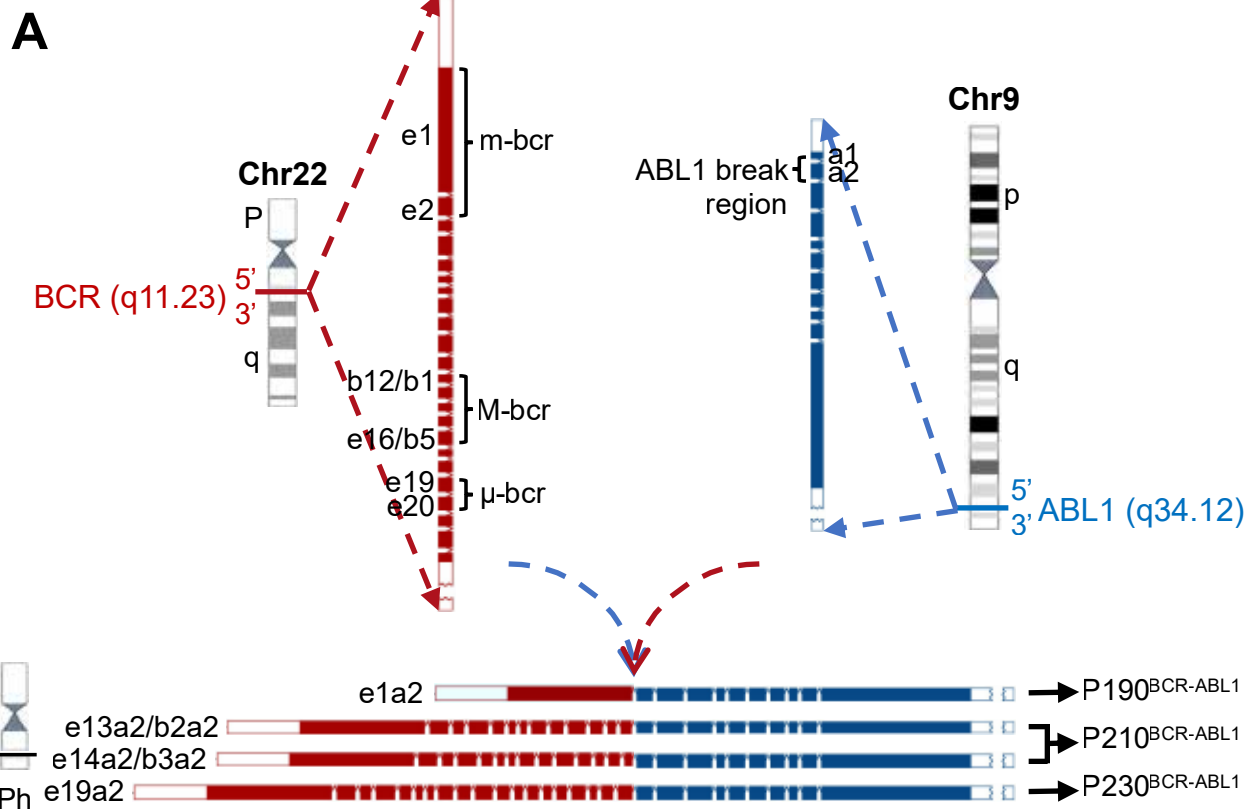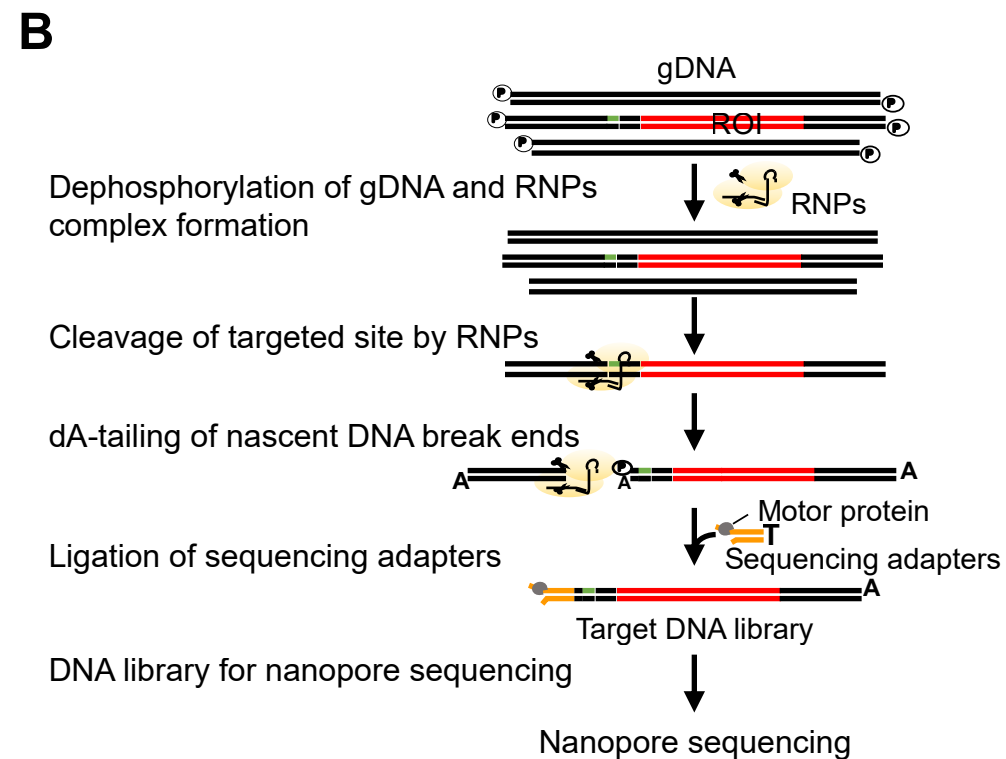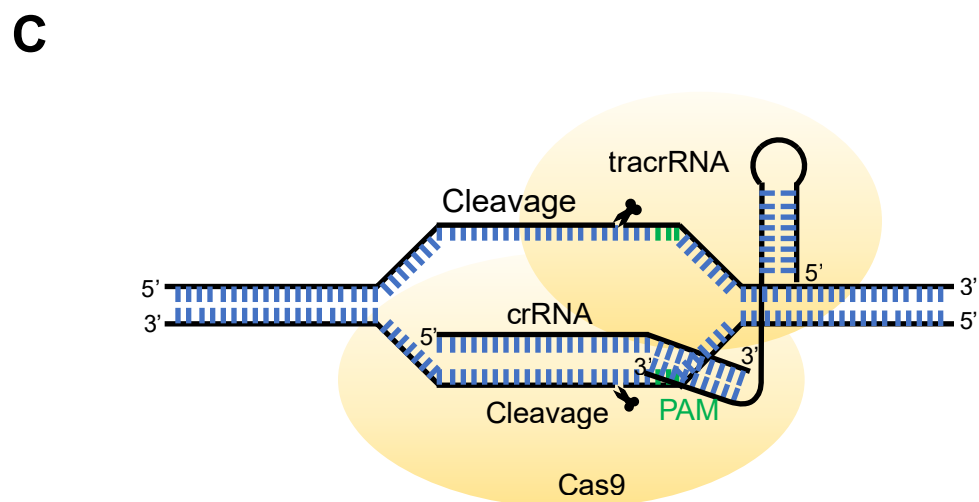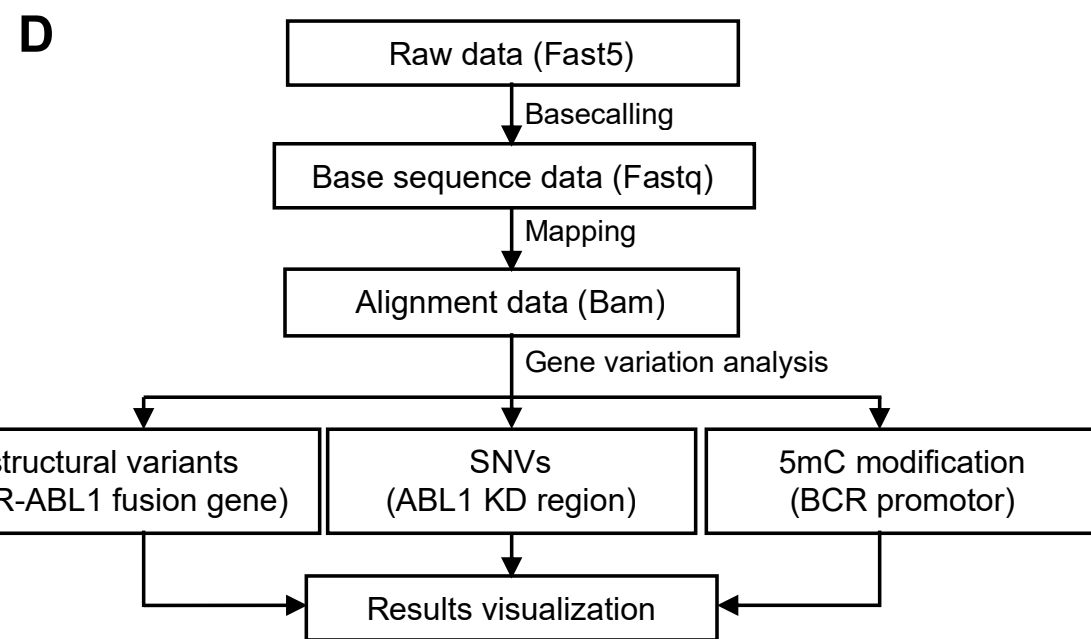

Supplement: Multimedia component 1 [file mmc1.pdf]

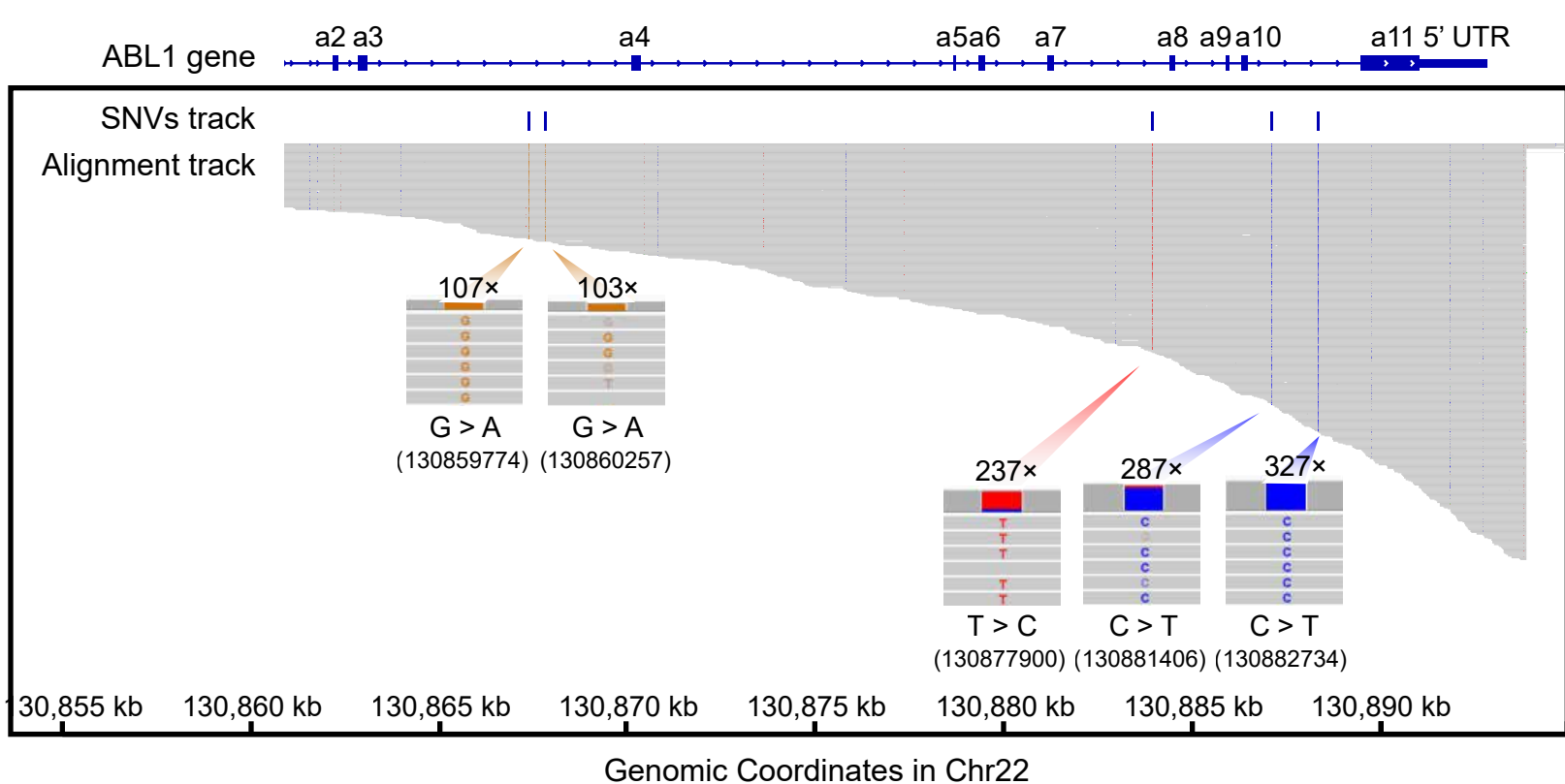

Supplement: Multimedia component 3 [file mmc3.pdf]

**A**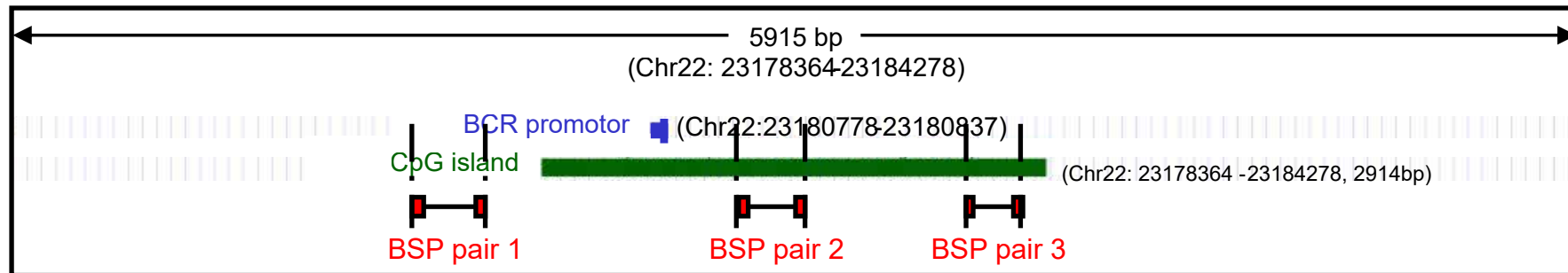**B**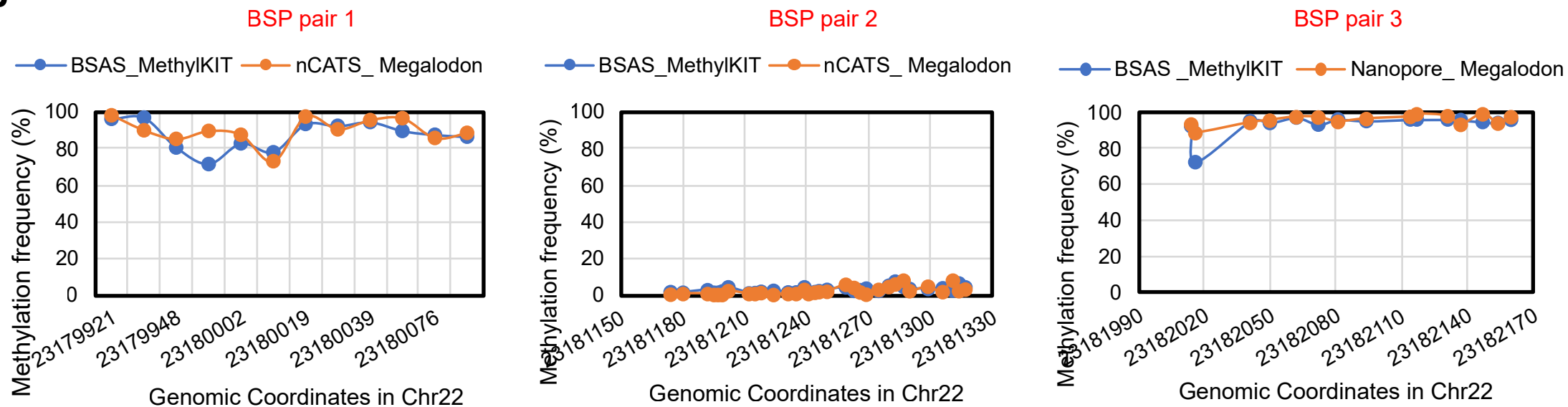

Supplement: Multimedia component 4 [file mmc4.pdf]

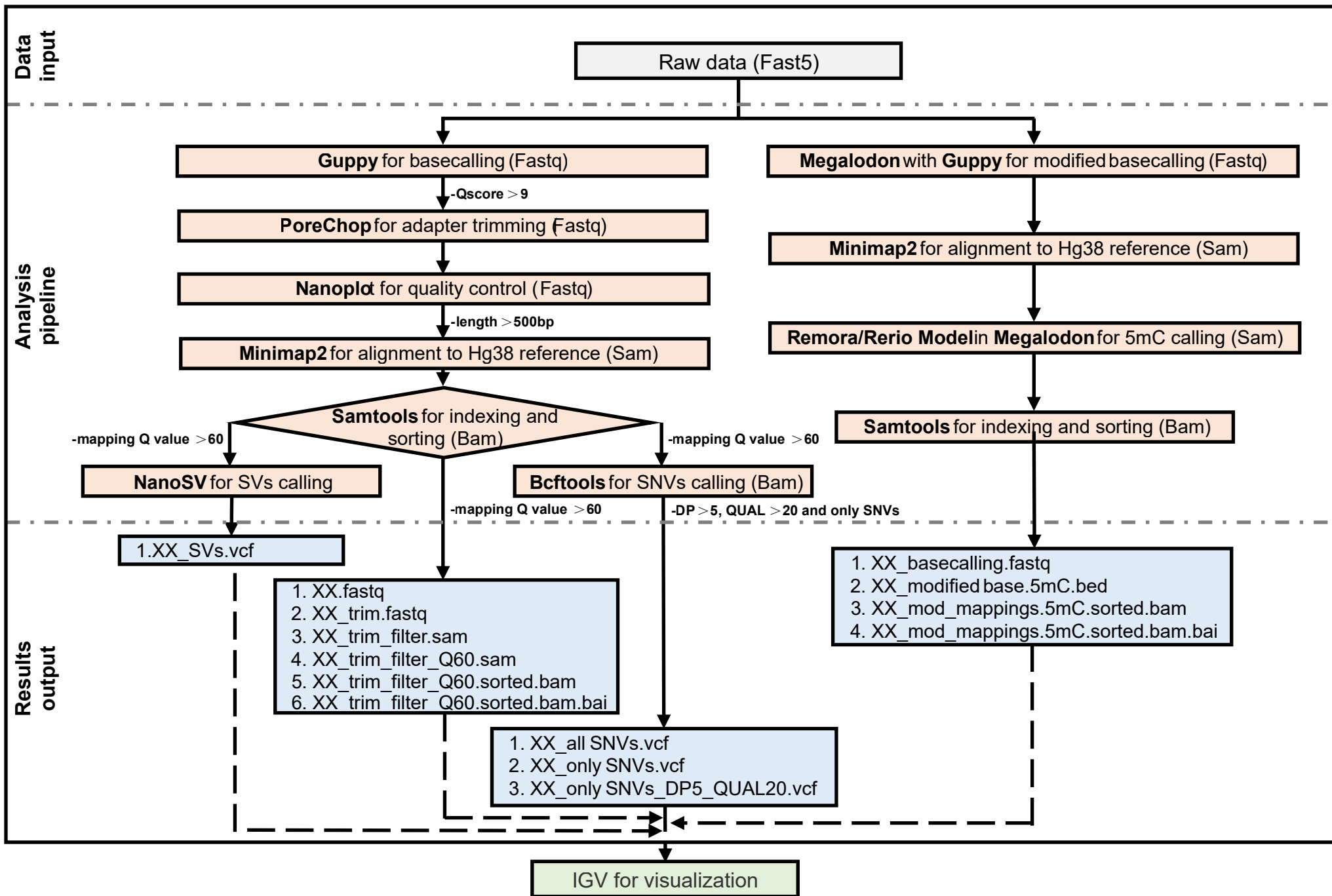

Supplement: Multimedia component 5 [file mmc5.pdf]

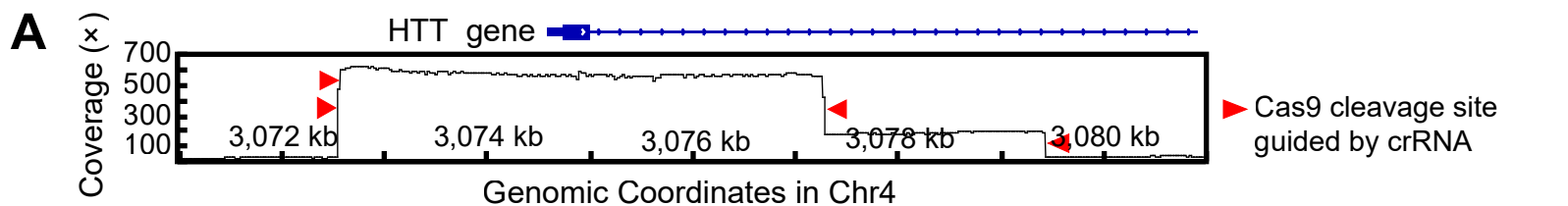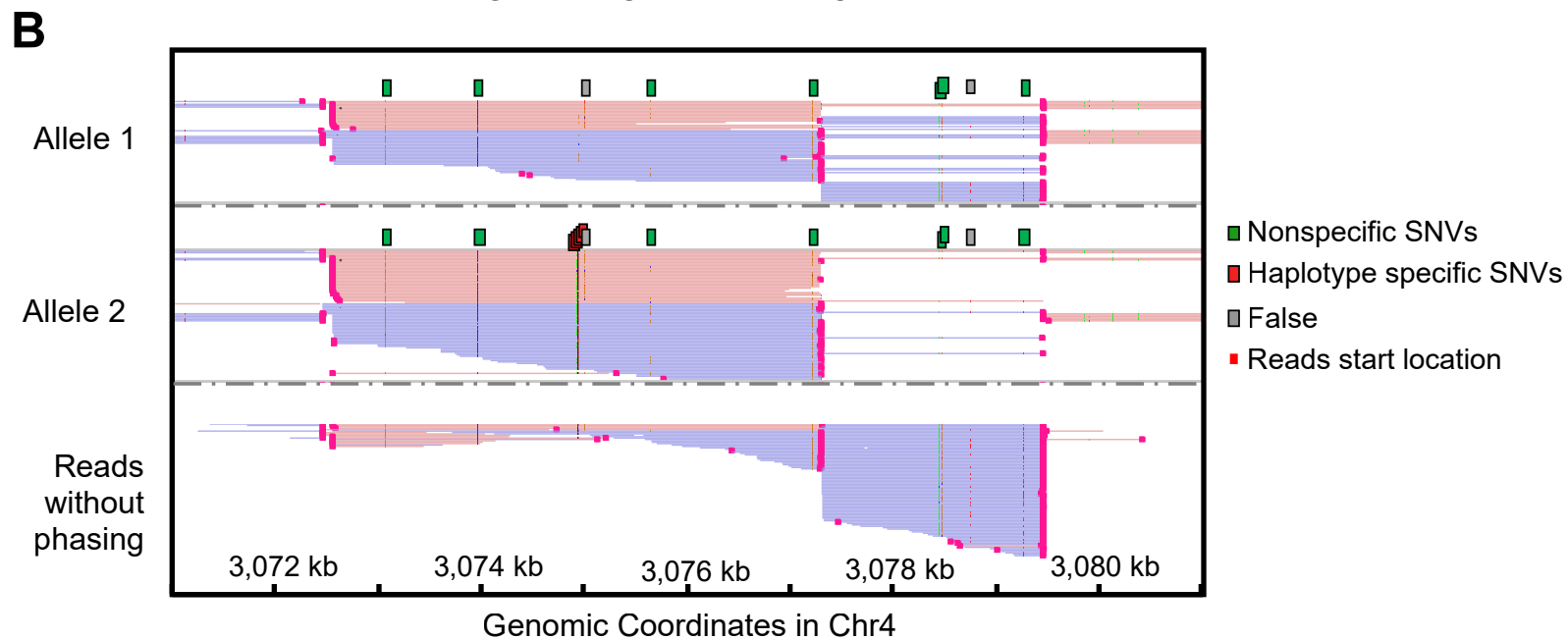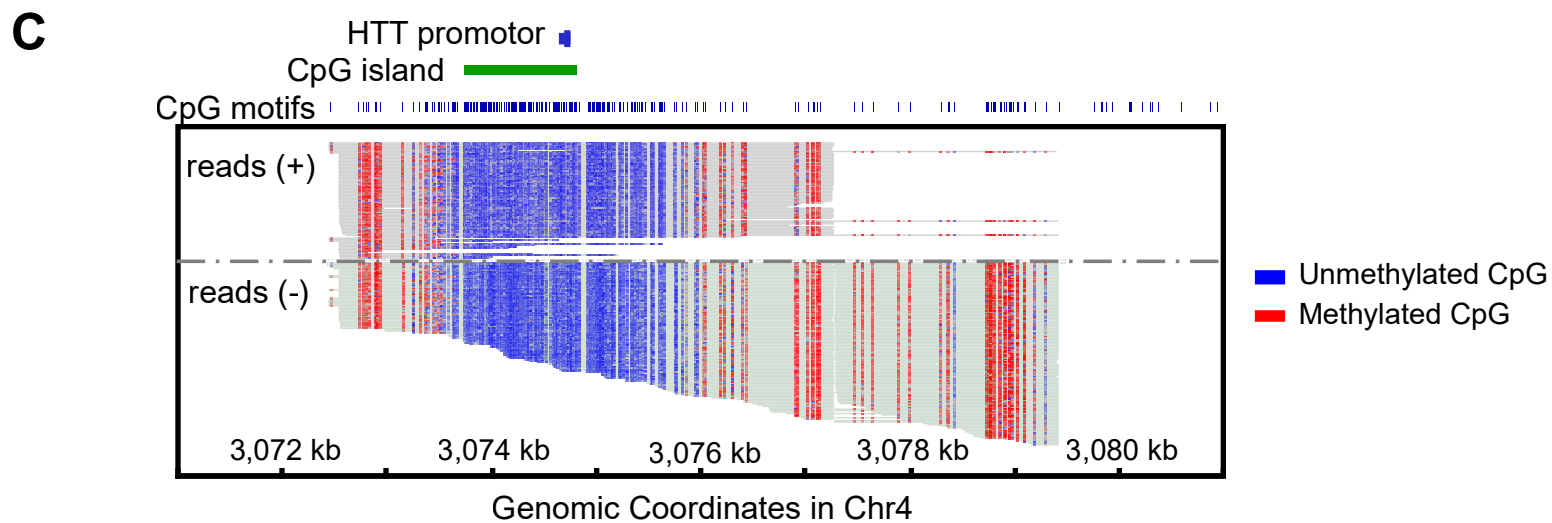

Supplement: Multimedia component 6 [file mmc6.pdf]
