# Supplementary material for: Simultaneous detection of methylation and genetic variations of BCR-ABL1 gene by nanopore Cas9-targeted sequencing
Source: Genes Dis. 2023 Dec 6;11(6):101190. doi: 10.1016/j.gendis.2023.101190 (PMC11327521; doi:10.1016/j.gendis.2023.101190)

A

crRNAs	BCR-23177705	BCR-23285487 + BCR-23286144	ABL1-130888808
Targeted ROIs	BCR promotor + BCR m-bcr	BCR M-bcr + BCR μ-bcr	ABL1 KD region
Number of on-target reads	**512**	**555**	**474**

B

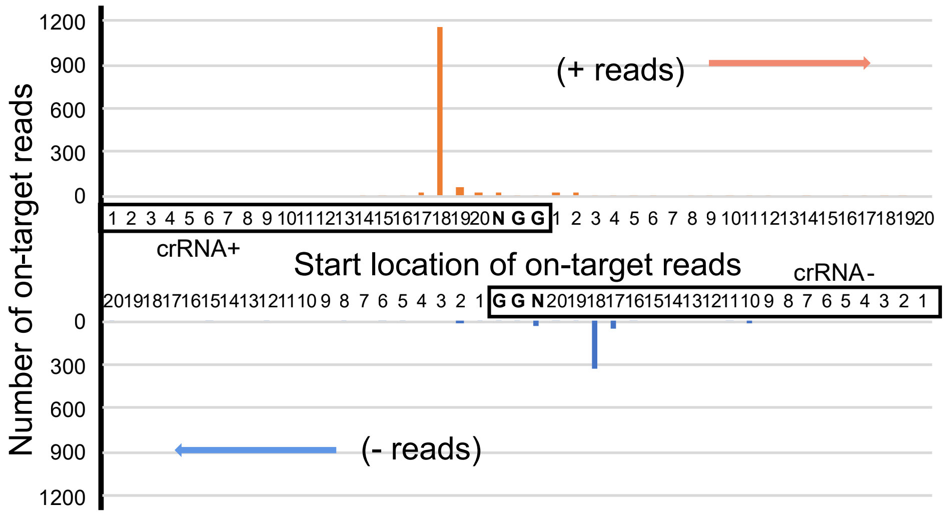

C

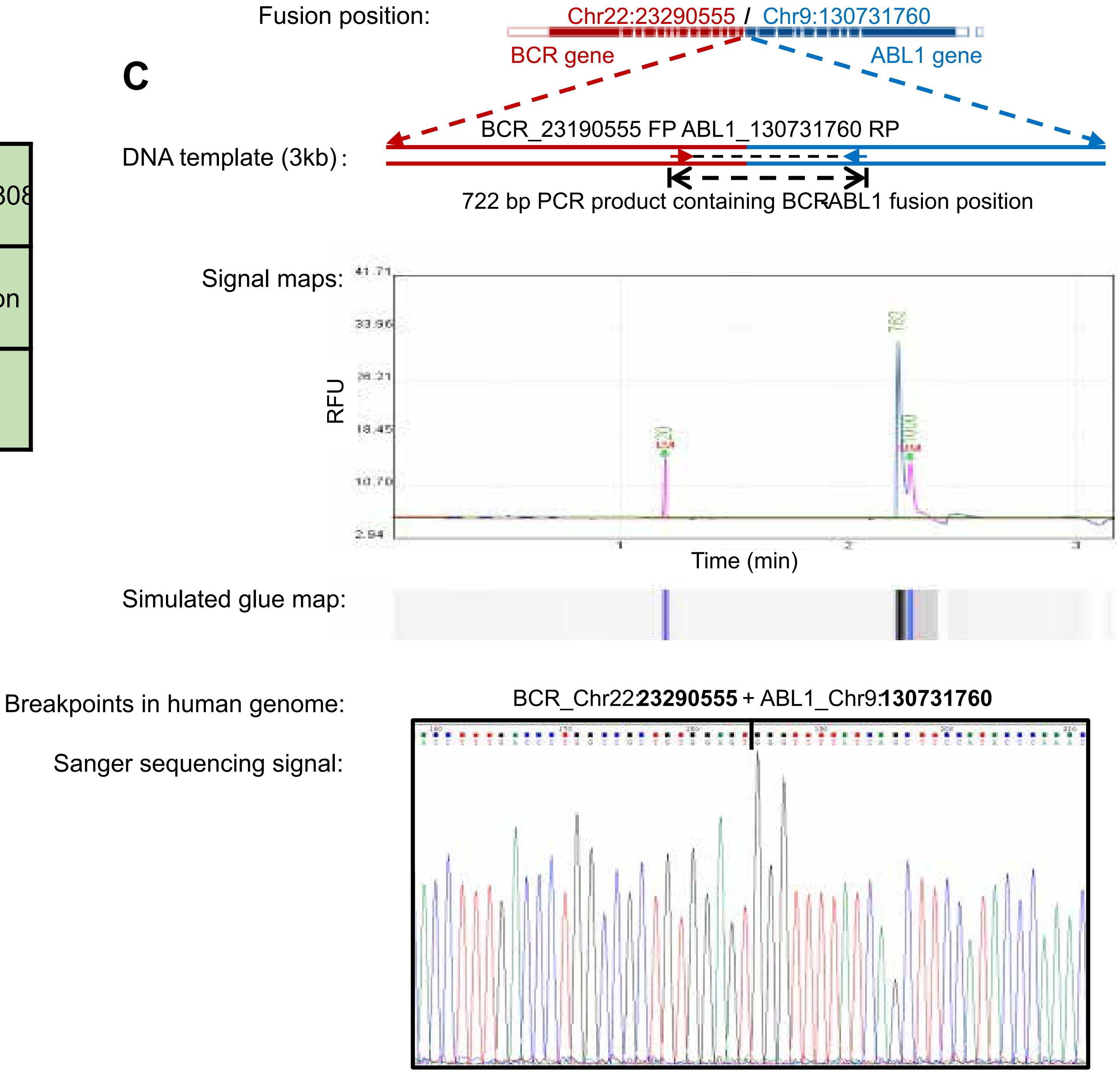

Supplement: Multimedia component 2 [file mmc2.pdf]
