# Supplementary material for: Simultaneous detection of methylation and genetic variations of BCR-ABL1 gene by nanopore Cas9-targeted sequencing
Source: Genes Dis. 2023 Dec 6;11(6):101190. doi: 10.1016/j.gendis.2023.101190 (PMC11327521; doi:10.1016/j.gendis.2023.101190)
Supplement: Multimedia component 14 [file mmc14.docx]

**Methods**

**Cell culture and gDNA extraction**

K562 cells were purchased from Wuhan Procell Life Science&Technology Co., Ltd. and cultured according to standard protocol. K562 cells were grown in RPMI 1640 medium supplemented with 10% fetal bovine serum (FBS) and 1% penicillin-streptomycin, and cultured in 37℃, 5% CO_2_ and humidity saturated incubator. K562 cells’ gDNA was extracted using the QIAamp DNA Blood Mini Kit (QIAGEN, 51106), and stored at -80℃ until use. Prior to performing the assay, the gDNA sample was quality controlled for concentration and fragment size using Qubit (ThermoFisher) and Qsep100 (Bioptic), respectively, which should have a concentration greater than 210 ng/μL and a fragment size around 10 kb.

**Design and synthesis of crRNA**

crRNA (IDT, custom) and tracrRNA (IDT, Catalog No. 1072532) were assembled as single guide crRNA (gRNA), which guided exonuclease Cas9 to cleave at ROI flanks. The online software “CHOPCHOP (<https://chopchop.cbu.uib.no/>)” was used to design crRNA, and the specific method referred to ONT official protocol of "Targeted, amplification-free DNA sequencing using CRISPR/CAS". A total of four crRNAs were designed to target ROIs in this study based on structural features of BCR-ABL fusion gene, including BCR_23177705 crRNA+ targeting the upstream region of BCR minor breakpoint cluster region (m-bcr) and BCR promoter, BCR_23285487 crRNA+ and BCR_23286144 crRNA+ targeting the upstream region of BCR main breakpoint cluster region (M-bcr), and ABL1_130888808 crRNA- targeting the downstream of ABL1 gene 3' end. The other four crRNAs used to enrich HTT promoter region (as in-run control) was obtained directly from the above ONT protocol. Sequence (20 bp guide sequence of crRNA + 3 bp NGG PAM) and targeting sites for all crRNAs were provided in supplementary Table S1.

**gRNA assembly and ribonucleoproteins (RNPs) incubation**

Lyophilized crRNAs and tracrRNAs were resuspended in IDTE (IDT, catalog no. 11-01-02-02) at a concentration of 100 µM, and then all crRNAs used for the same cleavage reaction were pooled in equal volumes to a total concentration of 100 µM. Both the crRNAs pool and tracrRNAs were denatured to gRNA duplexes at 95 °C for 5 minutes and cooled to room temperature such that both had a concentration of 10 μM. Ribonucleoprotein complexes (RNPs) were constructed by combining 30 pmol of annealed gRNA duplexes with 30 pmol of HiFi Cas9 Nuclease V3 (IDT, catalog no. 1081060) in 1× Reaction Buffer (ONT, catalog no. SQK-CS9109) at a final volume of 30 μL (concentration of 1 μM), incubated for 30 minutes at room temperature, then stored at 4 °C until use. Subsequent cleavage reactions with each gDNA sample were required to 10 μL, so any excess amount of RNPs may be stored at 4 °C for up to a week.

**Cas9 cleavage and library preparation**

The sequencing library preparation was followed by the protocol from ONT manufacturer’s instructions using the commercial kit (ONT, catalog no. SQK-CS9109). Briefly, before RNPs cleavage, input 1~10 μg gDNA (5μg recommended) was dephosphorylated in a total of 30 µL containing 24 µL of gDNA, 3 µL of Reaction Buffer and 3 µL of Phosphatase for 30 minutes at 37 °C in order to removing 5’ phosphates from arbitrary and untargeted DNA ends. The phosphates were heat inactivated at 80 °C for 2 minutes. When the dephosphorylated gDNA sample was allowed to return to room temperature, 10 µL of prepared RNPs was added to the sample tube along with 1 µL of Taq polymerase and 1 µL dATP. The reaction of 42 µL was mixed by gentle inversion and incubated at 37 °C for 30 minutes to enable Cas9 digestion and 72 °C for 5 minutes for dA-tailing addition by Taq polymerase. Following monoadenylation, the cleaved and dA-tailed gDNA sample was combined with 38 µL of adapter ligation mix for nanopore adapter ligation: 20 µL of Ligation Buffer, 10 µL of T4 Ligase, 5 µL Adapter Mix and 3 µL of Nuclease-free water (ThermoFisher, catalog no. AM9937), and incubated at room temperature for 10 minutes. Once completed, the ligation reaction (80 µL) was diluted with 1 volume of SPRI Dilution Buffer, and then cleaned up using 0.3× Ampure XP beads (48 µL) (Beckman Coulter, catalog no. A63881), washing twice on a magnetic rack with 250 µL Short Fragment Buffer. The washed beads absorbing adapter-ligated gDNA sample were eluted in 12~13 µL Elution Buffer for 30 minutes at room temperature. The supernatant, which contained targeted DNA library, was retained in a clean 1.5 ml Eppendorf DNA LoBind tube and incubated on ice until the flow cell was primed.

**Nanopore sequencing**

The DNA library was run on a R9.4 flow cell purchased from ONT, using the MK1B or GridION sequencer. Prior to loading the sequencing library, the flow cell needed to be primed via the priming port by adding 800 µL of priming solution mixed from one tube of Flush Buffer (ONT Flow Cell Priming Kit, catalog no. EXP-FLP002) and 30 µL of Flush Tether (ONT Flow Cell Priming Kit, catalog no. EXP-FLP002) and left to incubate for 5 minutes at room temperature. In parallel, the DNA library was prepared for nanopore sequencing by adding 37.5 µL of Sequencing Buffer (ONT Cas9 Sequencing Kit, catalog no. SQK-CS9109) and 25.5 µL of Loading Beads (ONT Cas9 Sequencing Kit, catalog no. SQK-CS9109). At the end of the incubation, the sequencing library was then loaded in a dropwise fashion from SpotON sample port after complete activation of the flow cell was performed by adding the remaining 200 µL of priming solution. The Sequencing device control, data acquisition and real-time basecalling were carried out by the MinKNOW software (v. 21.10.4).

**Nanopore sequencing data base-calling and bioinformatics analysis**

The FAST5 were converted to FASTQ sequencing reads by using the ‘high-accuracy’ basecalling model in Guppy 4.0.15. After nanopore adapters of the reads with Q-score > 9 were trimmed by PoreChop, length and quality control of the adapter-trimming reads were performed with Nanoplot for statistical plotting and the reads with length < 500bp were filtered by Filtlong. The filtered reads were aligned to the hg38 human reference genome using Minimap2. The produced SAM file with mapping score > 60 was compressed to BAM format with Samtools and simultaneously indexed and sorted for the analysis of genetic variation. Then, per-nucleotide coverage was determined after Bedtools conversion of BAM to BED format.

Since a single cut was used to excise the ROI, on-target reads were defined as those that aligned with the 20 bp of both flanks of crRNA site as the start location. The coverage depth of ROI was shown to be high on one side and low on the other.

Structural variants were detected from the BAM file with NanoSV using default parameters. The output result VCF file gave information directly on the breakpoints of BCR gene and ABL1 gene and can also be visualized in combination with BAM in IGV ([IGV: Integrative Genomics Viewer](https://igv.org/)).

Clair3, Bcftools and Freebayes were compared for SNVs detection. Due to the large number of InDels contained in the variant detection results in the generated VCF file, it needed to be rejected to remove interferences. In addition, we set Sequencing Depth (DP) and Mutation-calling Quality (QUAL) as the common lowest values, DP>10 and QUAL>20, respectively, with the exception of Clair3's QUAL filtering criteria were to remove low quality variant, which differed from the other two software. And because there were some low-frequency resistance mutations in ABL1 KD region, the Alter Frequency (AF) parameter was still set to the default value of 0. The resulting SNVs after filtering were annotated using SnpEff and annotation dataset used was dbSNP release 155. To enable identification of SNVs at the haplotype resolution, segregation of reads into parental alleles was performed with WhatsHap based on the haplotype-specific SNVs present in long reads.

Methylation calling on raw nanopore data was processed to produce high accuracy modified base (per-read and aggregated per-reference site) by Rerio 5mC basecalling models of Megalodon, using a single command with following parameters: ‘megalodon *XX.Fast5*/ --guppy-param "-d /home/dell/github/rerio/basecall_models/" --guppy-config res_dna_r941_min_modbases_5mC_CpG_v001.cfg --outputs basecalls mappings mod_mappings mods per_read_mods --reference /home/dell/genome/GRCh38.p12.genome.fa --mod-motif m CG 0 --devices 0 --processes 20 --mod-map-emulate-bisulfite --mod-map-base-conv C T --mod-map-base-conv m C --guppy-server-path /home/dell/github/ont-guppy/bin/guppy_basecall_server --output-directory ./*XX.Results*/ --write-mods-text’. The result can be visualized in the bisulfite mode of IGV.

**Bisulfite amplification sequencing (BSAS)**

Bisulfite treatment of the K562 gDNA with quality control was performed using EZ DNA Methylation-Gold Kit (Zymo, catalog no. D5005) according to the manufacturer’s protocol. To verify the methylation status of the CpG island containing BCR promotor by BSAS, three DNA fragment surrounding BCR promotor were amplified from the bisulfite-treated K562 gDNA with the following three pairs of Bisulfite Primer (BSP): BSP pair-1: 5’-TTAGGTTGTGAGGTGTGAGGAAT-3’ and 5’-ACCTCATATTCRGAAACAAAAACTA-3’; BSP pair-2: 5-GGTTAAGGAAAAGAAGAGTTATGAT-3’ and 5’-CTAACCTTACCRGAAA-AACCCT-3’; BSP pair-3: 5’-TTATTTTTAGYGAGGAGGAT-3’ and 5’-AARGCCCARGAT-AATAACCT-3’, where R is either a A or a G and Y is either a C or a T, all depending on the methylation status of a particular CG site. There are 13, 33 and 18 CG dinucleotides in these three fragments amplified by the three pairs of BSP, with lengths of 242 bp, 192 bp and 109 bp, respectively. For each PCR, 0.2 μM forward and reverse primers, 0.25 μL EX Taq HS (Takara, catalog no. RR006A), 2.5μL 10× Ex Taq Buffer (Takara, catalog no. RR006A), 2 μL dNTP Mixture (Takara, catalog no. RR006A), and 1.5 μL bisulfite-treated K562 gDNA template were used in a 25 μL total reaction volume. The PCR procedure consisted of 5 minutes at 95 °C for pre denaturation, 45 cycles of 15 seconds at 95 °C, 20 seconds at 58 °C and 20 seconds at 72 °C, and followed by a final extension at 72 °C for 10 minutes. The PCR products with correct bands were purified by gel extraction from a 1.5% gel using GeneJET Gel Extraction Kit (ThermoFisher, catalog no. K0691). Qualified purified PCR products were sequenced on Illumina platform using VAHTS Turbo DNA Library Prep Kit (Vazyme, catalog no. ND607-01) according to the manufacturer’s instructions.

**Methylation calling of BSAS data**

The FASTQ transformed from raw images of Illumina sequencing by basecalling were processed routinely such as Illumina adapters trimming, low-quality filtering and contamination removing, which resulted in high-quality sequence data. Depending on the C/T alteration, high-quality data can be mapped to the target genomic segment to obtain the localization information of the respective PCR products in human genome (hg38). Finally, Methylkit was used to analyze the above processed data to obtain the site information of methylated C.

**Statistical analysis**

The statistical method employed in this study was Pearson's correlation coefficient (P), which measured the degree of correlation between two variables X and Y with values between -1 and 1. A P value closer to 1 indicates a strong positive correlation between two variables; The closer the P value is to -1, the stronger the negative correlation between two variables; whereas a P value close to 0 indicates no correlation between the two variables. The statistical software used was Excel.

**Discussion**

BCR-ABL1 fusion gene is the mechanism of related leukemogenesis, and the occurrence of ABL1 KD mutations and abnormal 5mC modification of BCR promoter on this molecule is closely related to TKI resistance and disease progression. Here we successfully developed an nCATS method for simultaneously detecting the fusion breakpoints, KD mutations and promoter 5mC methylation of the BCR-ABL1 fusion gene.

During the design phase of this study, we designed a total of 4 crRNAs for targeting enrichment of native DNA fragments containing three molecular events of interest for nanopore sequencing to obtain the data containing long reads, deep sequencing, single-base resolution and modification information for these regions. The enrichment of the targeted DNA fragments was based on the orientation of the designed crRNA. Depending on the DNA strand of complementary pairing, crRNA was divided into two types: if the sequence of crRNA was complementary paired with the DNA negative strand while consistent with the DNA positive strand, it was used to enrich the DNA fragments downstream of its targeting position, and the strand for nanopore sequencing was the positive strand, called positive crRNA or crRNA+; conversely, it was called negative crRNA or crRNA-. This was due to the targeted DNA strand located on 5' side of crRNA forming a DNA-RNA hybrid with a 20 bp guide sequence of crRNA, so that the Cas9 protein was attached at the broken end, whereas the DNA fragment on the 3' side of the crRNA became exposed, thus enabling the preferential addition of a dATP and ligation of a sequencing adaptor by T/A. While performing the sequencing library, it is necessary to dephosphorylate the 5' end of gDNA before Cas9 cleaves the targeting sites of crRNAs, to prevent subsequent sequencing adaptors from ligating to arbitrary DNA ends. This treatment effectively removed a large number of non-targeted DNA fragments from sequencing, avoided the output of invalid sequencing data, and reduced the burden of subsequent data analysis. In addition, to ensure the success of the final nanopore sequencing, rigorous quality control of gDNA samples was required, as well as monitoring the sequencing full process (see methods section for details).

For BCR-ABL1 fusion assay, we designed three crRNAs+ located upstream of the three poorly spanning breakpoint regions in BCR gene, to ensure that nanopore sequencing was performed starting from the fusion head BCR gene with relatively conserved breakpoint regions and proceeding to the fusion tail ABL1 gene with a large span of breakpoints. This way both effectively circumvented the testing difficulties posed by fusion tail ABL1 gene with complex and variable breakpoints and enabled testing of some unknown fusion partners located in the tail. The nCATS detected the precise genomic coordinates of BCR and ABL1 breakpoints at a DNA-based level, which can be used for the finer classification of fusion subtypes and the analysis of specific motifs at the break sites. This was clearly superior to the commonly used qPCR detection kit that can only detect one known subtype of BCR-ABL1 fusion. And because BCR breakpoints were associated with patient prognosis and treatment response, the accuracy and superiority of nCATS established in this study in detecting BCR-ABL1 fusion gene is important in guiding the treatment of BCR-ABL1-positive patients.

Nanopore sequencing is based on neural network algorithms (RNN) for the basecalling of electrical signal alterations caused by approximately 4 to 6 randomly combined bases, which results in a high error rate of 5% to 10% with false-positive small insertions and deletions (InDels) being the most common. However, the error rate can be greatly reduced in nCATS by its high coverage characteristics to ensure the accuracy of detection of SNVs in ABL1 KD region. In addition, because we mainly focused on TKI resistance mutations occurring in ABL1 KD region, we only needed to analyze SNVs, not InDels, which further simplifies SNVs analysis. Despite the sequencing depth of ABL1 KD region appeared to be low on the 3' side and high on the 5' end, the analysis of nCATS data of BCR-ABL1 fusion gene in K562 cells’ gDNA showed that the lower site also reached about 80×, which was much higher than the lower sequencing DP filtering criteria (> 5×) of SNVs analysis tools (e.g., Bcftools or Clair3) that were weakly affected by the sequencing depth. So, even if the coverage of exon a2 (the leftmost part of ABL1 KD region) is low, about only 20x, we can still have reason to believe that the known TKI resistance mutations in the ABL1 KD region analyzed, even *de novo* TKI resistance mutations, are credible. The feature of long-read length in nCATS data is similarly advantageous in SNVs detection in the ABL1 KD region. The long reads targeted by ABL1_130888808 crRNA- completely covered of ABL1 KD region for detection of all SNVs occurring within the region. In addition, the long reads also enable phasing analysis based on haplotype-specific SNVs, which can facilitate the study of not only allelic differences and their effects on gene expression, function, and phenotype, but also the pathogenesis of genetic diseases.

The reads enriched by BCR_23177705 crRNA+ located upstream of BCR m-bcr also covered the CpG island within BCR promoter, and can be used for analysis of the methylation status of BCR promoter. Compared with traditional bisulfite-converted sequencing, nCATS exhibited superiority in both detection range and resolution, as shown by its ability to accurately determine the methylation status of each CG motif in the CpG island of approximately 2 kb at single-base resolution. And nCATS eliminates the need for bisulfite treatment of gDNA samples and subsequent PCR amplification, which not only reduces DNA degradation and the loss of bias and modification information from PCR amplification, but also greatly simplifies the experimental method and saves a lot of time. In addition, the CRISPR/Cas9 enrichment of DNA fragments that contain the BCR promoter could further ensure the accuracy and reliability of the detection results for 5mC modification. In terms of clinical implications, nCATS is a powerful technology for further unraveling the role of BCR promoter in the diagnosis and treatment of BCR-ABL1-positive leukemia, such as TKI resistance-related studies, the use of tumor molecular markers, and applications of methylating targeting drugs.

To facilitate the nCATS data analysis performed in this study, we developed a slick software package “Cas9-nanopore” for the analysis of structural variants, SNVs, and 5mC modifications in nCATS data. Cas9-nanopore combined three analysis pipelines for three genetic variants into a single software package, which enabled the raw FAST5 files to be processed with only a single command to directly output the results. Compared to the convoluted process of using multiple software packages, the one-step method of Cas9-nanopore greatly simplified nCATS data analysis and provided significant time savings. After analyzing the nCATS data of HTT gene targeted by four other crRNAs in the K562 cells’ gDNA, we validated the utility of Cas9-nanopore software. As can be seen, this software has good application prospects in analyzing tumor molecular markers such as genetic variations (SVS and SNVs) and epigenetic variations (5mC) in nCATS data.

In conclusion, this study implemented nCATS to detect BCR-ABL1 fusion gene and its KD mutations and promoter methylation status, which provided a more comprehensive and efficient test for BCR-ABL1-related hematological neoplasms. This will not only enable the diagnosis of CML and BCR-ABL1-positive ALL, but also, more importantly, enable the early identification of TKI resistance mutations occurring in the ABL1 KD region, so that treatment strategies can be developed or adjusted in a timely manner to implement precise targeted therapy for each individual. In addition, this study provides a streamlined platform that can be used by the basic science community to evaluate the 5mC modification of the BCR promoter. However, there are some limitations. The limitation of nCATS in sample throughput hindered its widespread application, as it cannot be multiplexed due to the lack of barcoding. In the future, multiplex nCATS method is needed to expand its wide application in scientific research and clinical settings by improving the sample throughput of nCATS.

**Captions of Supplementary Figure**

**Fig. S1 Schematic of nCATS procedure and classification of BCR-ABL1 fusion genes. (A)** The BCR-ABL fusion gene consists of a 5’ part of the BCR gene and a 3’ part of the ABL1 gene. The location of the translocation usually involves the fusion of intron 13 or 14 of BCR (in M-bcr) with a 140-kilobase (kb) region of ABL1 between exons 1a and 2a, so the fusion types of e13a2 and e14a2 (also known as b2a2 and b3a2) is the most common. Ph+: Philadelphia chromosome (t9;22) due to BCR-ABL1 fusion gene. M-bcr, main breakpoint cluster region; m-bcr, minor breakpoint cluster region; μ-bcr, the third breakpoint cluster region; UTR, untranslated region; Ph, Philadelphia chromosome. **(B)** Purified gDNA was dephosphorylated and then ribonucleoproteins (RNPs) assembled by crRNA, tracrRNA and Cas9 protein cleaved the target site to generate new DNA cuts, which can be ligated with sequencing adaptors in an A/T complementary manner after addition of a dA-tailing before loading target DNA library on the nanopore sequencer. gDNA, genomic DNA; ROI, region of interest. **(C)** The Cas9 protein cleaves the target site under the guidance of crRNA and the specification of a PAM motif (NGG). Theoretically, the cleavage site of Cas9 protein is generally located at the third base upstream of the PAM motif. **(D)** Brief process of data analysis, mainly including basecalling, alignment to reference genome, and downstream analysis of structural variants, SNVs, and 5mC modification. SNVs, single nucleotide variants; 5mC, 5-methylcytosine.

**Fig. S2 On-target analysis of the ROIs and verification of BCR-ABL1 fusion gene in K562 cells.** **(A)** Table showed targeted gene regions of interest and on-target reads enriched by the 4 crRNAs. **(B)** Start location distribution of on-target reads (corresponding to cleavage-site distribution of Cas9) targeted by the crRNAs used in the detection of K562 cells’ BCR-ABL1 fusion gene. The upper bar represented positively on-target reads (orange arrow), and the lower bar represented negatively on-target reads (blue arrow). **(C)** Three images were used to illustrate the validation of BCR-ABL1 fusion gene in K562 cells. The top is the designed primer pair of Polymerase Chain Reaction (PCR) based on the fusion site analyzed by the nCATS data of BCR-ABL1 fusion gene in K562 cells. The middle is the result of the full-automatic nucleic acid protein analysis system “Qsep100” for PCR products of the above primer pair. The final Sanger sequencing signal map showed the PCR products and the result of base sequence around fusion region was completely consistent with that of nCATS.

**Fig. S3 Analysis of single nucleotide variant (SNVs).** Visual representation of SNVs in ABL1 KD region of K562 cells detected by Bcftools. There were 5 annotated SNVs with rsID in ABL1 KD region (exon a2 to a11) from nCATS data of BCR-ABL1 fusion gene of K562 cells. UTR, untranslated region.

**Fig. S4 Verification of methylation status in BCR promoter of K562 cells by bisulfite amplification sequence (BSAS). (A)** Design of bisulfite sequencing primer (BSP) and verified DNA fragments characterized by high-low-high methylation.  **(B)** Comparison of methylation sequencing results between BSAS and BCR-ABL1 fusion gene nCATS data of the above three fragments.

**Fig. S5 Workflow of Cas9-nanopore software package.** The Cas9-nanopore software package consists of three bioinformatics analysis pipelines for identifying SVs, SNVs and 5mC modification. The output results file name of "XX_" can be modified according to experimental data. SVs, structural variants; SNVs, single nucleotide variant; 5mC, 5-methylcytosine.

**Fig. S6 The application of Cas9-nanopore software package for analyzing HTT gene sequencing data by nCATS. (A)** Coverage plot at a ROI containing the promoter of HTT gene from K562 cells’ gDNA with 2 crRNAs on both sides. **(B)** Annotated SNVs with rsID were analyzed by Cas9-nanopore from the nCATS data of the captured ROI, and the on-target reads were phased into two homologous alleles using WhatsHap. **(C)** Visualization of methylation analysis in and around the promoter of HTT gene from the nCATS data of K562 cells’ gDNA. ROI, region of interest.
